# The Effect of Nutritional Status in the Pathogenesis of Critical Illness Myopathy (CIM)

**DOI:** 10.3390/biology3020368

**Published:** 2014-05-30

**Authors:** Hannah Ogilvie, Lars Larsson

**Affiliations:** 1 Department of Neuroscience, Clinical Neurophysiology, Uppsala University, Uppsala, SE-751 85, Sweden; E-Mail: Hannah.ogilvie@neuro.uu.se; 2 Department of Biobehavioral Health, The Pennsylvania State University, University Park, PA 16802, USA

**Keywords:** nutrition, caloric intake, critical illness myopathy, intensive care

## Abstract

The muscle wasting and loss of specific force associated with Critical Illness Myopathy (CIM) is, at least in part, due to a preferential loss of the molecular motor protein myosin. This acquired myopathy is common in critically ill immobilized and mechanically ventilated intensive care patients (ICU). There is a growing understanding of the mechanisms underlying CIM, but the role of nutritional factors triggering this serious complication of modern intensive care remains unknown. This study aims at establishing the effect of nutritional status in the pathogenesis of CIM. An experimental ICU model was used where animals are mechanically ventilated, pharmacologically paralysed post-synaptically and extensively monitored for up to 14 days. Due to the complexity of the experimental model, the number of animals included is small. After exposure to this ICU condition, animals develop a phenotype similar to patients with CIM. The results from this study show that the preferential myosin loss, decline in specific force and muscle fiber atrophy did not differ between low *vs*. eucaloric animals. In both experimental groups, passive mechanical loading had a sparing effect of muscle weight independent on nutritional status. Thus, this study confirms the strong impact of the mechanical silencing associated with the ICU condition in triggering CIM, overriding any potential effects of caloric intake in triggering CIM. In addition, the positive effects of passive mechanical loading on muscle fiber size and force generating capacity was not affected by the nutritional status in this study. However, due to the small sample size these pilot results need to be validated in a larger cohort.

## 1. Introduction

The most frequent neuromuscular disorders underlying muscle weakness in intensive care unit (ICU) patients is critical illness myopathy (CIM). CIM is characterized by reduced muscle membrane excitability and a preferential loss of the molecular motor protein myosin. A number of different descriptive names have been given to this disorder, such as acute quadriplegic myopathy, thick filament myosin myopathy, acute myopathy of severe asthma and myopathy of intensive care [1,2]. In 1977, Macfarlane and Rosenthal [3] first reported a case of acute quadriplegia, affecting spinal nerve-innervated muscles, with intact sensory, cognitive and craniofacial muscle function [3].

Initially, CIM was thought to be a rare event in the ICU; we now know that neuromuscular dysfunction is found in up to 30% of the general ICU population and in 70%–80% of certain sub-groups [4,5,6]. The exact mechanisms underlying CIM remain unknown, although mechanical ventilation, sepsis, systemic corticosteroid administration and neuromuscular blocking agents have been proposed as factors triggering CIM [7]. The incomplete understanding of underlying mechanisms is, at least in part, related to insufficient diagnostic tools and the complex study of generalized muscle weakness in ICU patients, such as differences in underlying disease, age and gender. In addition, it has been impeded by a delay in the collection of muscle samples and preceded by several weeks after exposure to factors triggering a cascade of complex signaling pathways controlling protein synthesis and degradation.

Given the difficulty of controlled clinical observations, there is a compelling need for an experimental animal model which mimics the basic ICU conditions of long-term mechanical ventilation, sedation and muscle unloading: *i.e.*, absence of mechanical loading related to muscle contraction or weight bearing (mechanical silencing). The use of a unique experimental ICU model allows for detailed studies of skeletal muscle in mechanically ventilated, deeply sedated, pharmacologically paralyzed and extensively monitored rats for several days. Using this model, we have recently shown in a time-resolved analysis that complete mechanical silencing *per se* is a dominating factor triggering the preferential myosin loss, atrophy and loss of specific force (SF) in fast- and slow-twitch muscles and muscle fibers in limb skeletal muscles [7], while craniofacial muscles are spared or less affected [8]. Furthermore it is shown to induce a phenotype considered pathognomonic of CIM seen in ICU patients [9,10]. Furthermore it has been shown that passive loading in both experimental [11] and clinical studies [12] has a positive effect on muscle fiber function, strongly supporting the importance of early physical therapy and mobilization of deeply sedated and mechanically ventilated patients.

In addition to the possible factors triggering CIM listed above, insufficient nutrition/caloric intake is another factor that may play a substantial role in overall muscle loss and weakness in critically ill patients [13,14,15,16,17,18], however it remains unclear and of great debate as to whether artificial nutritional support benefits the outcome of critically ill patients [19]. Admission to the ICU often results in reduced energy and protein intake, increased energy expenditure and protein catabolism [14]. The aim of nutritional care is to minimize ‘acute disease-related malnutrition’; adequate nutritional care takes into consideration the interaction of actual disease, interventions and organ dysfunction with nutritional care [20]. Furthermore, it is suggested that the dramatic loss of lean body mass is affected by the nutritional care given to critically ill ICU patients. In fact, Puthucheary recently reported that increasing protein delivery was associated with increased muscle wasting [21]. Critical illness has been seen to evoke endocrine disturbances and a feeding-resistant hyper-catabolism, characterized by profound protein loss [22]. Furthermore, malnutrition is common among patients admitted to the ICU, an average of 37%–68% of patients are fed less than their nutritional requirement and exacerbated further by a prolonged stay [23,24].

### 1.1. Nutritional Guidelines

Several guidelines, observational studies and meta-analysis support the early administration of artificial nutrition; within 24–48 hours of ICU admission, of which the severely ill patients appear to benefit the most, but there are some discrepancies between different guidelines [19]; European Society for Clinical Nutrition and Metabolism (ESPN) [25] and The American Society for Parenteral and Enteral Nutrition (ASPEN) [26]. However, large interventional studies have been carried out comparing these two methods and have found there to be no benefit of early parenteral nutrition with regards to mortality and a clear disadvantage in resource use due to the duration of artificial ventilation and length of stay in the ICU [27,28]*.* The Early Parenteral Nutrition Completing Enteral Nutrition in Adult Critically Ill Patients (EPaNIC) trial showed that tolerating macronutrient deficit for 1 week in intensive-care units (late parenteral nutrition [PN]) accelerated recovery compared with early PN [19]. Further to this study in 2011 Hermans *et al.* carried out a sub-analysis to the EPaNIC trial finding that tolerating a substantial macronutrient deficit early during critical illness did not affect muscle wasting, but allowed more efficient activation of autophagic quality control of myofibers and reduced weakness and that in fact nutritional interventions may have an adverse effect on muscle whilst suppressing autophagy [27].

The aim of this study is to investigate the effects of the nutritional status in the pathogenesis of CIM by using a unique experimental animal model mimicking the ICU condition to determine whether nutritional status is driving the preferential myosin loss, fiber atrophy and loss of specific force in skeletal muscle, all of which are key characteristics of CIM. The results from this study show that nutritional status plays a minor etiological role compared with the mechanical silencing in driving the CIM phenotype. However, the technical complexity of the model and long duration of the experiments have limited the number of animals studied and these results need to be validated in future studies in a larger cohort.

## 2. Experimental Section

### 2.1. Animals

Nine female 300 g ± 5 g Sprague-Dawley rats were included in this study. A total of four rats were euthanized after a period of 0 days (serving as controls (C), no mechanical ventilation). Five animals underwent surgery to induce the experimental ICU condition (see below) [29,30] and remained in the mimicked ICU setting; anaesthetized, mechanically ventilated and treated with α-cobrotoxin (post‑synaptic neuromuscular blockade) for a 10–14 day period. Two animals were administered with the required caloric nutrition (eucaloric EC) and three with low caloric nutrition (low calorie (LC)).

The daily metabolic requirement calculations were based on energy consumption in an awake rat, receiving a combination of fat, protein and carbohydrates that were administered parenterally to the LC and EC animals. Detailed nutritional information is presented in Table 1. The ethical committee on human and animal research at Uppsala University, Uppsala, Sweden, approved all aspects of this study.

Table 1Detailed nutritional information**: **Low Caloric Intake. The appropriate intra-venous solution was administered. The total caloric intake was calculated to be approximately 11 kcal per day. (**a**) Example for a 300 g rat. (**b**) Detailed nutritional information: Eucaloric Intake: Corresponds to 41 kcal per day, for a 300 g rat; administered with an intra-venous solution with an increased nutritional content.

**Table:** (**a**)

100 mL vein infusion, low energy (11 kcal/day/kg bw)	Volumes
(0.6 mL/h)	
Lactated ringers	32 mL
Oxacillin	2.8 g
Glucose 50%	12.8 mL

**Table:** (**b**)

100 mL vein infusion, high energy (41 kcal/day/kg bw)	Volumes
(0.6 mL/h)	
Lactated ringers	32 mL
Oxacillin	2.8 g
Glucose 50%	31.6 mL
Vamin 114 mg/mL	9.4 mL
Intralipid 200 mg/mL	9.4 mL

### 2.2. Nutritional Information

Daily metabolic maintenance requirements of an awake rat can be calculated as:

Energy =112 × (rat weight in kg)^0.75^[kcal/d] [31]

This energy is recommended to be ingested in the form of fats, carbohydrates and proteins; broken down as: 20% fat (3 g), 75%carbohydrates (8.5 g), 5% protein (0.6 g).

Animals were maintained in a protein fluid balance: (1) Intra-arterial solution: Alfa Cobra toxin (Biotoxins, St. Cloud, Florida, FL, USA), Potassium, Oxacillin (Fresenius Kabi, Uppsala, Sweden, Heparin (Leo, Bellerup, Denmark), Ringers (Potassium Chloride, Calcium Chloride, Sodium Chloride, Lactate, Fresenius Kabi), Water; (2) Intra-venous solution: Oxacillin (Fresenius Kabi), Ringers, Water, Glucose (Glucose, Vamin, Fresenius Kabi) and Intralipid, (Fresenius Kabi).

In both instances, the LC and EC caloric groups were given the same intra-arterial solution, the intra-venous solution differs depending on caloric intake and individual rat’s body weights.

### 2.3. Experimental ICU Model

The experimental model has previously been described in detail [29,30] and modified to optimise studies of skeletal muscle [7,32]. Briefly: (1) Electrocardiogram (ECG) electrodes were implanted subcutaneously; (2) An aortic catheter (28-gauge Teflon) was inserted via the left carotid artery to record arterial blood pressure; (3) A 0.9 mm Renathane catheter was threaded into the left jugular vein to administer parenteral nutrition; (4) Three subcutaneous electroencephalogram (EEG) needle electrodes were placed into the skull above the right and left temporal lobes, and a third reference electrode was placed on the neck; (5) The rat was placed on a heating pad to maintain body temperature; temperature was measured by a vaginal thermistor and servo-regulated at 37 °C; (6) A silicone cannula was inserted in the urethra to continuously record urine output. In no case did animals show any signs of infections or septicemia.

### 2.4. Passive Loading

The left leg (loaded leg) of the LC (n = 3) and EC (n = 2) groups were activated 12 hours per day, using a mechanical lever arm that produced a continuous passive maximal ankle joint flexion-extension of 180° at a speed of 13.3 cycles per minute and the right leg served as the unloaded internal control. Controls underwent no mechanical loading on either leg.

To analyze the function of myofilament proteins in cells with an intact filament lattice, but without the confounding effects of intercellular connective tissue or protein heterogeneity of whole muscle or multicellular preparations, we measured the force generating capacity of single muscle fibers.

### 2.5. Muscle Tissue Preparation and Fiber Membrane Permeabilization

The tibialis anterior (TA), extensor digitorum longus (EDL), gastrocnemius and soleus muscles were dissected from the loaded left leg and the unloaded right leg immediately after euthanasia. One half of the soleus and EDL muscles were quickly frozen in liquid propane cooled by liquid nitrogen, and stored at −160 °C for future analyses. The other half of the soleus and EDL muscle were dissected into bundles. Specimens were placed in relaxing solution at 4 °C and bundles of approximately 50 fibers were dissected free and tied with surgical silk to glass capillary tubes at slightly stretched lengths. They were then treated with skinning solution (relaxing solution containing glycerol; 50:50 v/v) for 24 hours at 4 °C, after which they were transferred to −20 °C. The bundles were detached from the capillary tubes and treated with sucrose, a cryoprotectant, within 1–2 weeks for long-term storage, snap frozen in liquid nitrogen-chilled propane and stored at −180 °C [33].

### 2.6. Single Muscle Fiber Experimental Procedure and Specific Force Measurements

On the day of experiment, bundles were de-sucrosed; transferred to a 2.0 M sucrose solution for 30 minutes and subsequently incubated in solutions of decreasing sucrose concentrations (1.5–0.5 M) and finally kept in a skinning solution at −20 °C.

A single fiber was removed from the muscle bundle and was placed between two connectors, at a length of 1 to 2 mm long. One connector leads to a force transducer (model 400A, Aurora Scientific Inc., ON, Canada), and the other to a lever arm system (model 308B, Aurora Scientific) [34,35]. The two extremities of the fiber were tightly attached to the connectors as previously described [34]. The apparatus was mounted on the stage of an inverted microscope (model IX70; Olympus, Hitech Inc., PA, USA). The sarcomere length was set to 2.65–2.75 µm by adjusting the overall segment length [35] and controlled during the experiment using a high-speed video analysis system (model 901A HVSL, Aurora Scientific). The diameter of the fiber between the connectors was measured through the microscope at a magnification of ×320 with an image analysis system prior to the mechanical experiments. Fiber depth was measured by recording the vertical displacement of the microscope nosepiece while focusing on the top and bottom surfaces of the fiber. The focusing control of the microscope was used as a micrometre. Cross-sectional area (CSA) was calculated from the diameter and depth, assuming an elliptical circumference, and was corrected for the 20% swelling that is known to occur during skinning [34]. Diameter and depth were measured at three different locations along the length of each fiber and the mean was considered a representative of cell dimensions.

For the mechanical recordings, relaxing and activating solutions (in mM) contained 4 Mg-ATP, 1 free Mg^2+^, 20 imidazole, 7 EGTA, 14.5 creatine phosphate and KCl to adjust the ionic strength to 180 mM and pH to 7.0*.* The concentrations of free Ca^2+^ were 10^−9.00^ M (relaxing solutions) and 10^−4.50^ M (activating solution), expressed as pCa (*i.e.*, −log10 [Ca^2+^]). Apparent stability constants for Ca^2+^-EGTA are corrected for temperature (15 °C) and ionic strength (180 mm). The computer program Fabiato [36] is used to calculate the concentration of each metal, ligand and metal–ligand complex. At 15 °C, immediately preceding individual activations, the fiber is immersed for 10–20 seconds in a solution with a reduced Ca^2+^-EGTA buffering capacity. This solution was identical to the relax solution except that the EGTA concentration is reduced to 0.5 mm, which results in a more rapid attainment of steady-state force during subsequent activations.

Force was measured by slacking the fiber once steady-state isometric force was reached at pCa 4.5. Seven slacks were performed for each fiber and maximum force was calculated as the difference between the maximal steady-state isometric force in activating solution and the resting force measured in the same segment while in relaxing solution. Maximal force production was normalized to CSA (specific force *P*_0_/CSA).

For all the contractile measurements a strict acceptance criteria is applied. First, the sarcomere length was checked during the experiments, using a high-speed video analysis system (model 901A HVSL; Aurora Scientific). A muscle fiber was accepted and included in the analyses if the sarcomere length of a single muscle fiber changed by <0.10 μm between relaxation and maximum activation, and maximal force changed by <10% from first to seventh activation [34].

### 2.7. Myosin Heavy Chain (MyHC) Isoform Expression and Myosin:Actin Ratios

After mechanical recordings, each skinned fiber was placed in sample buffer (7.43 mL distilled water, 2.1 mL Glycerol, 1.4 mL 10% SDS, 1.75 of 0.5 M Trisbuffer pH 6.8, 0.32 mL bromophenol blue, 32.4 mg Dithiothreitol, 1 mL Leupeptin) in a plastic micro-centrifuge tube and stored at −180 °C for subsequent electrophoretic analysis [37,38]. MyHC isoform composition of fibers was then determined by 6% SDS-PAGE. The acrylamide concentration was 4% (wt/vol) in the stacking gel and 6% in the running gel, and the gel matrix included 30% glycerol. Sample loads were kept small (equivalent to approximately 0.05 mm of fiber segment) to improve the resolution of the myosin heavy chain bands (types I, IIa, IIx and IIb). Electrophoresis was performed at 120 V for 20–22 hours with a Tris–glycine electrode buffer (pH 8.3) at 10 °C (SE 600 vertical slab gel unit, Hoefer Scientific Instruments). The gels were silver-stained and subsequently scanned in a soft laser densitometer (Molecular Dynamics, Sunnyvale, CA, USA) with a high spatial resolution (50 μm pixel spacing) and 4096 optical density levels.

Soleus and EDL 10-μm cryo-sections were dissolved in 100 μL urea buffer (8 M; 120 g urea, 38 g thiourea,70 mL H2O, 25 g mixed bed resin, 2.89 dithiothreitol, 1.51 g Trizma base, 7.5 g SDS) after centrifugation and heating (90 °C for 2 minutes). The samples were run on 12% SDS-PAGE and gel bands corresponding to actin and myosin were identified and quantified as previously described [10]. These values were used to determine myosin:actin protein ratios.

### 2.8. Statistical Analysis

Means and standard error of the mean was calculated according to standard procedures. One way-analyses of variance (ANOVA) were used when comparing multiple groups and *p* < 0.05 was considered statistically significant.

## 3. Results and Discussion

### 3.1. Body and Muscle Weight

A significant decline in body weight between pre and post experiments were observed in both the low caloric (LC; *p* < 0.05) and eucaloric (EC; *p* < 0.01) groups (Table 2). There was no significant difference in body weights between controls and pre-body weights in the LC and EC groups. In parallel with the decline in body weight, significantly lower muscle weights were observed between controls and the two experimental (LC and EC) groups (Table 2). In both experimental groups (LC and EC), passive mechanical loading had a sparing effect of muscle weight on the loaded (left) compared to the unloaded (right) side (Table 2).

**Table 2:** Shows pre and post total body and individual muscle weights in all groups. In the LC and EC groups, the left leg was mechanically loaded by passive ankle joint flexion’s-extensions 12 hours per day. Body weights decreased from pre to post experiment in the LC (*p* < 0.05) and EC groups (*p* < 0.01).

**Group**	**BW (g)**		**TA (mg)**		**EDL (mg)**		**GAST (mg)**		**SOL (mg)**	
	Pre	Post	Left	Right	Left	Right	Left	Right	Left	Right
Controls (0 days) n = 4	297 ± 23	297 ± 23	592 ± 10	598 ± 12	148 ± 6	148 ± 6	1690 ± 39	1692 ± 40	132 ± 3	135 ± 3
LC (10, 10 and –14 days) n = 3	295 ± 21	215 ± 20	330 ± 30	250 ± 20	95 ± 7	74 ± 6	780 ± 30	740 ± 20	100 ± 8	70 ± 3
EC (10 days) n = 2	290 and 308	219 and 223	342 and 384	319 and 376	101 and 109	88 and 105	876 and 953	790 and 900	85 and 101	71 and 64

Control (0 days), LC (10 days) and EC (10, 10 and 14 days) groups, BW: body weight, TA: Tibialis Anterior, EDL: extensor digitorum longus, GAST: gastrocnemius, SOL: soleus. Values are means ± SEM.

### 3.2. Myosin:Actin Ratios

**Figure 1:**
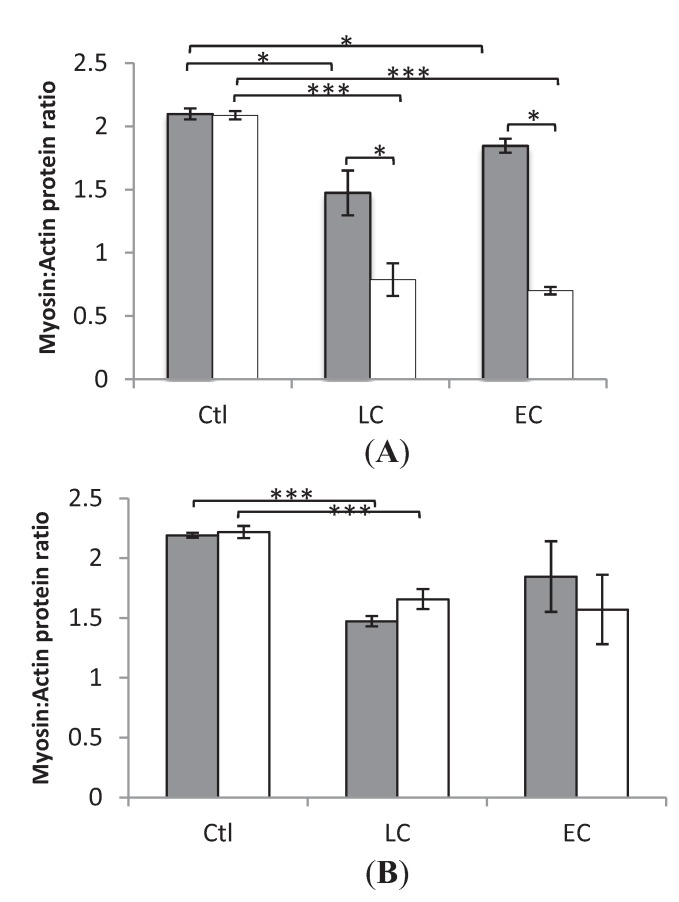
Average myosin:actin ratios. (**A**) Soleus (**B**) EDL. In the individual groups controls (0 Days) (Ctl), LC 10 days (LC), EC 10–14 days (EC), loaded (grey) and unloaded (white) limbs. * indicates statistically significant difference (*p* < 0.05) *** *p* < 0.001 between the unloaded and loaded leg of Ctl, EC and LC. Values are means ± SEM.

In both the loaded and unloaded legs, myosin:actin ratios did not differ significantly in either those who received LC or EC nutrition. However, between Ctl and LC in Soleus and EDL, loaded and unloaded, there was a statistical significance (* *p* < 0.05 and *** *p* < 0.001), as well as in soleus, loaded and unloaded between Ctl and EC. Furthermore there was a statistically significant difference (*p* < 0.05) between the unloaded and loaded leg of those who received LC and EC in the slow-twitch soleus but not in the fast-twitch EDL (Figure 1).

### 3.3. Muscle Fiber Size and Specific Force in Muscle Fibers Expressing Fast and Slow Myosin Heavy Chain (MyHC) Isoforms

A detailed analysis of fiber CSA and force generating capacity (maximum force, normalized to muscle fiber CSA), *i.e.*, specific force, was carried out at the single muscle fiber level. A total of 80 muscle fibers in the control and 120 in both the EC and LC groups fulfilled the criteria for acceptance (see Methods) and were analyzed; 100 soleus fibers expressing the type I MyHC isoform and 100 EDL fibers expressing IIx or IIb MyHC isoforms were included in the experimental groups. Muscle fiber CSA and SF measurements were carried out in both the loaded and unloaded leg of each animal.

**Figure 2:**
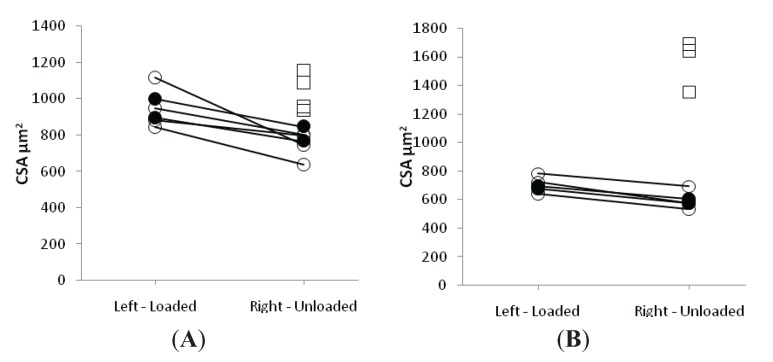
Average single muscle fiber area in the soleus (A) and the EDL (B) in the loaded and unloaded limbs in the control (squares), LC (open circles) and EC groups (filled circles).

**Figure 3:**
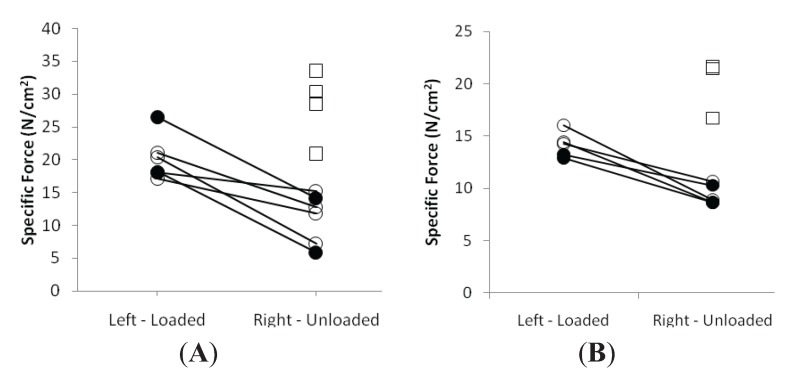
Average single muscle fiber specific force in the soleus (**A**) and the EDL (**B**) in the loaded and unloaded limbs in the control (squares), LC (open circles) and EC groups (filled circles).

In both LC and EC groups, passive loading had a significant positive effect on both muscle fiber size (*p* < 0.001) and force generating capacity (*p* < 0.001) irrespective of muscle fiber MyHC isoform expression (Figure 2 and Figure 3). In both LC and EC groups, muscle fiber CSA and SF at the end of the experimental period were significantly lower (*p* < 0.001), irrespective of muscle fiber MyHC isoform expression (Figure 2 and Figure 3). Loading appeared to have a stronger effect on both muscle fiber size and specific force in the soleus muscle fibers compared with the EDL fibers in accordance with the stronger effect of the passive loading on the myosin:actin ratio in the slow-twitch soleus than in the fast twitch EDL (Figure 2 and Figure 3).

In the loaded soleus muscle, no difference was observed between the EC and LC group in the muscle fiber CSA and SF (Figure 2 and Figure 3). In the unloaded soleus, on the other hand, a small but statistically significant difference (*p* < 0.05) was observed in fiber CSA between EC and LC groups (Figure 2A). The biological significance of this finding is questioned and it is assumed to represent a type 1 statistical error.

In the EDL muscle, unloaded and loaded muscles showed no statistical difference between the EC and LC group in CSA or SF (Figure 2 and Figure 3).

### 3.4. Discussion

The major findings from this study is that low *vs.* eucaloric parenteral nutrition had no significant effect on the size or force generating capacity (specific force) in single muscle fibers and myosin:actin ratios were not affected in either the slow-twitch soleus or the fast-twitch EDL muscles in animals exposed to a mimicked ICU setting over a period of 10–14 days. Irrespective of caloric intake, animals developed a phenotype considered characteristic of CIM, *i.e.*, a preferential myosin loss, fiber atrophy and decreased specific force. Furthermore, the caloric intake did not have an additional effect on the improvement in muscle fiber size or force in response to passive mechanical loading. Thus, indicating that caloric intake is of less importance compared with e.g., mechanical signaling in the pathogenesis of CIM in ICU patients. Although these results are of significant scientific importance and clinical interest, it is important that these results be validated in a larger cohort in future studies.

The reduced preferential myosin loss in response to passive mechanical loading, irrespective of caloric intake, in the slow-twitch soleus but not in the fast-twitch EDL is in accordance with our previous observations [11]. This muscle specific effect is suggested to be secondary to the loading effect on both protein synthesis and degradation, together with the higher protein turnover rate in slow- *vs.* fast-twitch limb muscles [11].

Skeletal muscle serves as a major protein reservoir in the critically ill patients. During prolonged critical illness muscle strength and function are compromised, which in turn can have a detrimental effect on recovery and more specifically in weaning patients from the ventilator [22]. The treatment of critically ill ICU patients has improved rapidly in recent years, resulting in improved survival rate and reduced duration of the ICU treatment. The nutritional care of ICU patients has on the other hand, not improved at the same rate and there is a lack of consensus as to what is considered to be the optimal feeding regime in critically ill ICU patients [16,39,40]. In some studies, a higher caloric intake was associated with a poorer outcome, including mortality and infections [21,41,42,43]. In 2003, Krishnan showed that feeding fewer calories, between 33% and 66% of the estimated energy needs, was associated with improved clinical outcomes compared with patients who received closer to 100% of goal calories. [42] Others in an aggregate of 90 septic trauma or burn patients, have shown that the net balance between protein synthesis and protein breakdown is improved with protein intakes up to 1 to 1.5 g/kg/day, whereas any further increase in protein intake is wasted in oxidative pathways [44,45,46]. Furthermore, Streat and co-workers found that although malnutrition in the critically ill is a recognised predictor of a poor patient outcome, nutritional support in the ICU has proved powerless in preventing a loss of lean body tissue [21,47].

In a previous experimental and clinical study from our group [7,11,12], we have shown that the removal of both weight bearing and activation of contractile proteins, *i.e.*, ‘mechanical silencing’ of skeletal muscle is a dominating factor triggering the specific myopathy (CIM) associated with the ICU condition. Furthermore, the loss of muscle mass and function can be attenuated by early mechanical loading [11,12] supporting early physical therapy in immobilized ICU patients [48]. The results from this study demonstrate that the loading effect was similar in the LC and EC groups, supporting the strong effect of mechanical signaling in the development and intervention of CIM and being of superior importance compared with caloric intake.

Non-depolarizing post-synaptic neuromuscular blockade has been suggested to be an important factor triggering CIM, but in our previous studies using a porcine ICU model we have not observed a significant difference in muscle structure and function in pigs exposed to mechanical ventilation and deep sedation with or without neuromuscular blockade [49].

### 3.5. Study Limitations

The unique experimental model used in this project mimics the ICU condition well. Unfortunately, this model is technically demanding and involves extensive monitoring 24 hours per day during the 10–14 day experimental period. This has limited the number of animals included in the LC and EC groups, which we acknowledge and suggest that a confirmatory study is needed. Thus, small differences in muscle size and function related to caloric intake may have gone undetected in this study. However, we suggest from the present results that such differences are negligible compared with the effects of mechanical silencing in inducing the CIM phenotype as well as the positive effects caused by passive mechanical loading. Furthermore, the most appropriate type and timing of feeding for ICU patients, *i.e.*, parenteral *vs.* enteral, has been debated for many years. In our model parenteral feeding was the means used to administer nutrition, but enteral nutrition is the preferred route of administration for critically ill patients [26,50], however there is a significant ongoing debate concerning this. Nevertheless, an average of 37%–68% of patients are fed less than their nutritional requirements with enteral nutrition and need supplemental parenteral feeding [23,24]. Thus, there is an impending need for further investigation and the development of knowledge into the effects of nutritional supplementation on muscle mass and on the regulation of muscle contraction at the single muscle fiber level; however this is beyond the scope of this study.

## 4. Conclusions

Although artificial nutrition plays an important role for energy metabolism in critically ill ICU patients, the present results show that caloric intake is not an important factor in triggering CIM or in the improvement associated with passive mechanical loading during the ICU condition.
